# HCN2 channels: a potential therapeutic target for orofacial neuropathic pain after trigeminal nerve injury

**DOI:** 10.22514/jofph.2026.013

**Published:** 2026-01-12

**Authors:** Toru Yamamoto, Tomoaki Ujita, Yurie Sato-Yamada, Takako Ichiki, Naotaka Kishimoto, Miho Terunuma, Kenji Seo

**Affiliations:** ^1^Division of Dental Anesthesiology, Faculty of Dentistry & Graduate School of Medicine and Dental Sciences, Niigata University, 951-8514 Niigata, Japan; ^2^Center for Advanced Oral Science, Faculty of Dentistry & Graduate School of Medicine and Dental Sciences, Niigata University, 951-8514 Niigata, Japan; ^3^Department of Oral Biochemistry, Faculty of Dentistry & Graduate School of Medicine and Dental Sciences, Niigata University, 951-8514 Niigata, Japan

**Keywords:** Hyperpolarization-activated cyclic nucleotide-gated channel, Rat, Neuropathic pain, Orofacial, Trigeminal

## Abstract

Hyperpolarization-activated cyclic nucleotide-gated (HCN) channels have recently emerged as promising targets for the treatment of neuropathic pain. This study investigated the potential involvement of HCN2 channels in the development of trigeminal neuropathic pain following peripheral nerve injury. Infraorbital nerve chronic constriction injury (ION-CCI) model was adopted to rats, and head withdrawal thresholds (HWT) to mechanical stimulation were assessed pre- and postoperatively, as well as after pharmacological intervention. In the trigeminal ganglion (TG), intracellular cyclic adenosine monophosphate (cAMP) and cytoplasmic protein kinase A (PKA) levels were quantified by Enzyme-Linked Immunosorbent Assay (ELISA), while *Hcn2* mRNA expression was evaluated by quantitative Polymerase Chain Reaction (qPCR). Immunohistochemical analysis was performed to assess phosphorylated cAMP response element-binding protein (pCREB) expression in the TG and HCN2 expression in infraorbital nerve (ION) axons. In the TG, cAMP and pCREB levels were elevated, whereas cytoplasmic PKA and *Hcn2* mRNA levels were reduced. Axonal HCN2 expression was increased in CCI rats. On day 14, HWT was significantly reduced following CCI but was ameliorated by local administration of the HCN channel blocker ivabradine at the site of axonal injury. Collectively, these findings suggest that CCI-induced alterations in cAMP-PKA-pCREB signaling promote HCN2 accumulation in injured axons, thereby contributing to the development of orofacial neuropathic pain following peripheral nerve injury.

## 1. Introduction

Peripheral nerve injuries represent a significant clinical challenge in dental practice, particularly after procedures such as third molar extractions, implant placements, and orthognathic surgeries. Damage to branches of the trigeminal nerve can lead to persistent neuropathic pain, characterized by spontaneous pain, hyperalgesia, and allodynia, severely impairing patients’ quality of life. Despite advancements in surgical techniques and perioperative care, effective strategies to prevent and treat dental neuropathic pain remain limited.

Neuropathic pain arises from aberrant excitability and spontaneous activity in damaged sensory neurons. Among the various ion channels implicated in this maladaptive neuronal behavior, hyperpolarization-activated cyclic nucleotide-gated (HCN) channels have emerged as critical contributors. HCN channels generate an inward current (Ih) upon hyperpolarization and play an essential role in regulating neuronal resting membrane potential and repetitive firing. In particular, the HCN2 isoform is highly expressed in nociceptive neurons, where it serves as a key determinant of pathological excitability [1].

Recent studies have demonstrated that pharmacological inhibition of HCN channels, particularly with ivabradine, produces promising analgesic effects in both preclinical models of neuropathic pain [2]. This emerging evidence underscores the need for further investigation into the molecular mechanisms underlying neuropathic pain and positions HCN2 as a promising therapeutic target for the development of novel, mechanism-based analgesic interventions [3, 4]. HCN2 channels expressed in the dorsal root ganglion (DRG) and trigeminal ganglion (TG) have been implicated in both inflammatory and neuropathic pain conditions [5, 6, 7]. However, the precise mechanisms by which HCN2 contributes specifically to trigeminal neuropathic pain remain unclear. In particular, it is not yet known whether peripheral nerve injury modulates intracellular signaling pathways, such as the cAMP-PKA-pCREB axis, within the TG, or whether it induces axonal accumulation of HCN2 in the trigeminal nervous system.

Therefore, the aim of this study was to investigate the involvement of HCN2 in trigeminal neuropathic pain using a rat model of infraorbital nerve chronic constriction injury (ION-CCI), focusing on both behavioral responses and molecular changes in the trigeminal ganglion and injured axons.

## 2. Materials and methods

### 2.1 Animals

All experimental procedures were approved by the Animal Research Committee of Niigata University (approval number: SA01042) and performed in accordance with the guidelines of the International Association for the Study of Pain (revised 2002) and the Guide for the Care and Use of Laboratory Animals (eighth edition, National Research Council of the National Academies). Male Sprague Dawley rats (6 weeks old; The Jackson Laboratory, Japan) were housed in pairs and maintained in a 12/12-h light/dark cycle at approximately 23 °C with free access to food and water. The number of animals used and their suffering were minimized in all experiments, following the 3Rs principle.

### 2.2 Neuropathic pain model

We used an infraorbital nerve chronic constriction injury (ION-CCI) model [8]. Sixty-three rats were randomly divided into two groups and anesthetized by intraperitoneal injection of 2.5 mg/kg butorphanol, 0.375 mg/kg medetomidine, and 2 mg/kg midazolam. The ION (left side) of 33 rats was partially ligated with two 4-0 silk ligatures (2 mm apart). In 30 sham-operated rats, the ION was exposed but not ligated.

### 2.3 Enzyme-linked immunosorbent assay (ELISA)

Fourteen days after ION-CCI, intracellular cyclic adenosine monophosphate (cAMP) and cytoplasmic protein kinase A (PKA) levels in the TG were quantified using a Parameter™ cAMP Assay (KGE012B, R&D Systems, Minneapolis, MN, USA) and a Rat Protein Kinase-A ELISA Kit (MBS1600249, MyBioSource, San Diego, CA, USA) for quantitative detection. The whole TGs were harvested under deep anesthesia and promptly homogenized using an ultrasonic cell disruptor (QSONICA, QSonica LLC, Newtown, CT, USA) for subsequent cAMP analysis and by hand using hand-homogenizers (Biomasher® II, Nippi, Inc., Tokyo, Japan) for cytoplasmic PKA according to the manufacturers’ instructions.

### 2.4 Immunohistochemistry

Fourteen days after ION-CCI, TGs and ION axons were harvested under deep anesthesia. The deeply anesthetized rats were transcardially perfused with 0.02 M phosphate-buffered saline (PBS; pH 7.2) and then with 4% paraformaldehyde in 0.1 M phosphate buffer (pH 7.2). TGs and ION axons were carefully harvested and fixed with 4% paraformaldehyde in 0.1 M phosphate buffer for at least 48 h. After being soaked in 20% sucrose in PBS overnight for cryoprotection, the tissues were embedded in Tissue-Tek OCT compound (#4583, Sakura Finetechnical, Tokyo, Japan) and frozen at −80 °C. Frozen sections were cut transversely into 20-µm-thick sections using a cryostat (CM1850, Leica Biosystems, Nussloch, BW, Germany).

The expression of pCREB in the TGs and HCN2 in axons (at the injured site of the infraorbital nerve in the CCI group and the corresponding site in the sham group) were examined using immunohistochemistry. The sections were incubated for 24 h with a primary antibody (rabbit anti-pCREB; MA5-11192, 1:1000, Invitrogen, Waltham, MA, USA; mouse anti-HCN2; ab84817, 1:1000, Wako, Tokyo, Japan), washed, and incubated for 1 h with a secondary antibody (Alexa fluor 594 for pCREB, 488 for HCN2; 1:1000; Thermo Fisher Scientific, Waltham, MA, USA). Images were captured using a fluorescence microscope (BZ-X800, Keyence, Osaka, Japan). The TG and axonal sections exhibiting the highest immunoreactivity, along with the two immediately adjacent sections, were selected for quantification of pCREB puncta and integrated density of HCN2. The number of pCREB-positive puncta was counted, and the integrated density of HCN2 immunofluorescence—including at the axonal constriction injury site—was measured by a blinded investigator using ImageJ (NIH, Bethesda, MD, USA). All analyses were performed on images captured at consistent magnification, exposure settings, angles, and anatomical locations across samples. The measurements from these sections were then averaged to obtain a representative value for each individual rat.

### 2.5 Reverse transcription quantitative real-time polymerase chain reaction (RT-qPCR)

Fourteen days after ION-CCI, TGs were harvested under deep anesthesia, and total RNA was immediately isolated using an RNeasy kit (#74104, QIAGEN, Hulsterweg, Venlo, Netherlands) and stored at −80 °C until use. qPCR was performed using a qPCR Master Mix kit (A6001, Promega, Madison, WI, USA) and a Quant Studio3 real-time PCR machine (Thermo Fisher, Waltham, MA, USA) according to the manufacturers’ instructions. The Ct values were normalized to β-actin, and fold changes were determined using the ΔCt method. The gene-specific PCR primers were: *Hcn2* F; 5ʹ-GGACCATCGGGAAGAAGATGTA-3ʹ, R; 5ʹ-GCTGAGATCATGCTGAACCTTG-3ʹ, and β-actin F; 5ʹ-CTTGCAGCTCCTCCGTCGCC-3ʹ, R; 5ʹ-CTTGCTCTGGGCCTCGTCGC-3ʹ.

### 2.6 Behavioral test

The mechanical sensitivity of the whisker pad, which is innervated by the ION, was evaluated using von Frey filaments (Semmes-Weinstein von Frey Anesthesiometer, Muromachi Kikai, Tokyo, Japan) before surgery as a baseline and fourteen days after ION-CCI or sham surgery. On day 14, rats received a local injection of ivabradine (0.5 mg diluted in 0.5 mL saline) around the injured site of the ION under temporary sevoflurane anesthesia. The von Frey tests were conducted immediately before the injection and again 30 minutes after the injection under awake conditions. All behavioral tests were conducted by a blinded investigator.

### 2.7 Statistical analysis

Data are expressed as mean ± standard deviation (SD). For behavioral experiments, two-way analysis of variance (ANOVA), followed by a multiple comparison test were used. The two factors assessed were time and intervention group. Unpaired *t*-tests were used to analyze normally distributed outcomes from protein assays and immunohistochemistry. Statistical analyses were performed using GraphPad Prism 9 (GraphPad, San Diego, CA, USA). A *p*-value < 0.05 was considered for statistical significance. A priori power analysis was performed using G*Power (version 3.1, Heinrich Heine University, Düsseldorf, NRW, Germany) with α = 0.05 and power (1 − β) = 0.8. The analysis indicated that more than 10 animals per group would be required to detect a medium effect size (Cohen’s *d* = 0.6–0.8). For each experiment, the final sample size was determined with careful consideration of the 3Rs principle, aiming to minimize animal use while maintaining statistical validity. This was guided by previous studies employing similar behavioral and immunohistochemical endpoints.

## 3. Results

### 3.1 ION-CCI facilitates cAMP-PKA-pCREB signaling and alters *Hcn2* mRNA levels in TG

From ELISA and qPCR analysis, the cAMP and pCREB levels in the CCI group were significantly higher (*p* = 0.026, *p * < 0.001, respectively) than in the sham group, while the cytoplasmic PKA and *Hcn2* mRNA levels in the CCI group were lower (*p* = 0.041, *p* = 0.007, respectively) than in the sham group (Fig. 1A–D).

**Fig. 1.** ION-CCI facilitated cAMP-PKA-pCREB signaling and altered *Hcn2* mRNA expression in TG. (A) The normalized concentration of cAMP in TG cells from the sham group and CCI group. *p = 0.026, unpaired *t*-test, n = 4 and 6, respectively. (B) The normalized concentration of cytoplasmic PKA in TG from the sham group and CCI group. *p = 0.041, unpaired *t*-test, n = 8 and 6, respectively. Note that decreased cytoplasmic PKA in TG of CCI rats suggests that increased cAMP activated PKA and then the activated PKA translocated from the cytoplasm into the nucleus. (C) Immunohistochemistry for pCREB (red color puncta) in TG from the sham group and CCI group. Scale bar = 200 µm. ****p  < 0.001, unpaired *t*-test, n = 4 and 5, respectively. (D) The normalized expression of *Hcn2* mRNA in TG cells from the sham group and CCI group. **p = 0.007, unpaired *t*-test, n = 6 per group. CCI, Chronic Constriction Injury; TG, trigeminal ganglion; cAMP, cyclic adenosine monophosphate; PKA, protein kinase A; pCREB, phosphorylated cAMP response element-binding protein.

### 3.2 ION-CCI increases HCN2 channel levels in injured axons and ivabradine blocking of peripheral HCN channels reverses ION-CCI-induced mechanical hyperalgesia

In the immunohistochemistry analysis, the integrated density of HCN2 immunofluorescence in axons was significantly higher in the CCI group than in the sham group (*p* = 0.003; Fig. 2A). In the behavioral tests, a significant difference in head withdrawal threshold (HWT) was observed between the CCI-Ivabradine and Sham-Ivabradine groups after surgery at the pre-injection time point, indicating that CCI decreased the thresholds (*p * < 0.001). Within the CCI-Ivabradine group, a significant difference in HWT was observed between pre- and post-injection, indicating that ivabradine reversed the thresholds (*p * < 0.001). In contrast, no significant difference in threshold was observed within the Sham-Ivabradine group (*p* = 0.880) (Fig. 2B).

## 4. Discussion

This study demonstrated that ION-CCI altered cAMP-PKA-pCREB signaling and *Hcn2* mRNA expression in the TG, and increased HCN2 protein levels in injured axons.

Emerging evidence indicates that cyclic nucleotide signaling pathways, such as those mediated by cAMP, play critical roles in both neuropathic and inflammatory pain. These pathways have been demonstrated in various animal models and are implicated in multiple pro-nociceptive mechanisms, including the activation of HCN channels [9, 10]. Elevated cAMP levels activate PKA, which subsequently translocates from the cytoplasm to the nucleus. In the nucleus, activated PKA promotes the production of phosphorylated cAMP response element-binding protein (pCREB), thereby facilitating the transcription of target genes. Accordingly, we investigated whether cAMP-PKA-pCREB signaling was altered following ION-CCI.

Based on our findings of altered cytoplasmic cAMP and PKA levels, as well as increased pCREB expression in the TG after CCI, we initially hypothesized that *Hcn2* mRNA levels would also be upregulated in the TG. However, contrary to our expectation, *Hcn2* mRNA expression was reproducibly decreased, which is consistent with previous reports examining the dorsal root ganglia [11].

It has been demonstrated that HCN channels accumulate within the sciatic nerve following constriction injury [12]. In addition to HCN channels, sodium channels [13] have also been shown to accumulate at nerve injury sites via axonal transport. Based on these findings, we next hypothesized that upregulated HCN2 channels produced in the trigeminal ganglion (TG) might be transported to the injured axonal site through axonal transport, a notion supported by our observations. Notably, ivabradine does not cross the blood-brain barrier and is excluded from the central nervous system [14], suggesting that the behavioral effects observed in this study are attributable to the blockade of peripheral HCN channels.

A previous report shows that this convergence leads to the formation of ectopic foci of action potential generation, ultimately contributing to the development of neuropathic pain following nerve injury [13]. In conclusion, increased HCN2 levels in injured axons may contribute to ION-CCI-induced orofacial hyperalgesia in rats (Fig. 2C), and blocking HCN2 channels in peripheral nociceptive neurons alleviates pain-related behaviors. Clinically, the involvement of HCN channels may explain why pain control using sodium channel blockers alone is insufficient in some cases [15]. Given that ivabradine had no effect on acute mechanical or thermal pain thresholds in control rats, and that HCN channels are not involved in normal acute pain processing [5], our findings suggest a potential clinical application: the combined use of sodium channel blockers with selective HCN2 inhibitors for the treatment of trigeminal neuropathic pain.

**Fig. 2.** ION-CCI increased HCN2 channel expression in injured axon and blocking peripheral HCN channels by ivabradine reversed ION-CCI-induced mechanical hyperalgesia. (A) The integrated density of HCN2 immunofluorescence in axons was significantly higher in the CCI group compared with the sham group (**p = 0.003, unpaired *t*-test; n = 4 per group). Scale bar = 500 µm. The area outlined by the dotted line indicates the region of HCN2-positive accumulation in the axon (proximal to the constriction site). (B) Among the groups, a significant difference in head withdrawal threshold was observed between the CCI-Ivabradine and Sham-Ivabradine groups after surgery at pre-injection time point, meaning that CCI decreased the thresholds (*p < 0.001, repeated measurement two-way ANOVA followed by Šídák’s multiple comparisons test). Within the CCI-Ivabradine group, a significant difference in head withdrawal threshold was observed between pre-injection and post-injection of ivabradine, meaning that ivabradine reversed the thresholds (^†^*p * < 0.001, repeated measurement two-way ANOVA followed by Tukey’s multiple comparisons test). Within the Sham-Ivabradine group, no significant difference in the threshold was observed between pre-injection and post-injection of ivabradine (*p* = 0.880, repeated measurement two-way ANOVA followed by Tukey’s multiple comparisons test). CCI-Ivabradine (n = 6), Sham-Ivabradine (n = 4), respectively. (C) A schema of axonal accumulation of HCN2 channel in the injured axon. CCI, Chronic Constriction Injury; HCN, Hyperpolarization-activated cyclic nucleotide-gated; TG, trigeminal ganglion; pCREB, phosphorylated cAMP response element-binding protein.

The present findings may have important implications for dental clinical practice. Trigeminal nerve injury can occur as a complication of various dental procedures, such as third molar extraction, implant placement, orthognathic surgery, and endodontic treatment. These injuries may lead to persistent orofacial neuropathic pain, for which effective treatment options remain limited. Our results suggest that axonal accumulation of HCN2 and the associated activation of cAMP-PKA-pCREB signaling may play a crucial role in the pathophysiology of such conditions. Understanding this mechanism could inform the development of novel, targeted therapies—such as selective HCN2 inhibitors—for the management of chronic orofacial pain of dental origin.

## 5. Limitations

The quantification method used in the immunohistochemical analysis of the present study may not entirely eliminate the possibility of bias. Ideally, analyzing entire tissue sections would have been preferable to further minimize selection bias.

In the present study, HCN1 was not investigated. Therefore, potential interactions between HCN1 and HCN2 warrant further examination in future studies. Because ivabradine inhibits all HCN isoforms (HCN1–4) equally [16], the future development and application of selective HCN2 blockers would be desirable.

In addition, only male rats were used in this study. Previous reports have indicated sex differences in pain mechanisms [17]. Moreover, differences between the trigeminal ganglion (TG) and dorsal root ganglion (DRG) have also been reported [12, 18]. Thus, future studies should investigate the roles of both HCN1 and HCN2 in the trigeminal system using both male and female animals to provide a more comprehensive understanding.
